# Novel Epigallocatechin-3-Gallate (EGCG) Derivative as a New Therapeutic Strategy for Reducing Neuropathic Pain after Chronic Constriction Nerve Injury in Mice

**DOI:** 10.1371/journal.pone.0123122

**Published:** 2015-04-09

**Authors:** Xavier Xifró, Laura Vidal-Sancho, Pere Boadas-Vaello, Carlos Turrado, Jordi Alberch, Teresa Puig, Enrique Verdú

**Affiliations:** 1 Grupo de Investigación de Anatomía Clínica, Embriología, Neurociencia y Oncología Molecular (NEOMA), Departamento de Ciencias Médicas, Facultad de Medicina, Universitat de Girona (UdG), Girona, Spain; 2 Departament de Biologia Cel·lular, Immunologia i Neurociències, Facultat de Medicina, Universitat de Barcelona, Barcelona, Spain; 3 Institut d’Investigacions Biomèdiques August Pi i Sunyer (IDIBAPS), Barcelona, Spain; 4 Centro de Investigación Biomédica en Red sobre Enfermedades Neurodegenerativas (CIBERNED), Madrid, Spain; 5 Laboratorio de Química Médica, Departamento de Química Orgánica I, Facultad de Ciencias Químicas, Universidad Complutense de Madrid (UCM), Madrid, Spain; Rutgers University, UNITED STATES

## Abstract

Neuropathic pain is common in peripheral nerve injury and often fails to respond to ordinary medication. Here, we investigated whether the two novel epigallocatechin-3-gallate (EGCG) polyphenolic derivatives, compound **23** and **30**, reduce the neuropathic pain in mice chronic constriction nerve injury (CCI). First, we performed a dose-response study to evaluate nociceptive sensation after administration of EGCG and its derivatives **23** and **30**, using the Hargreaves test at 7 and 21 days after injury (dpi). We daily administered EGCG, **23** and **30** (10 to 100 mg/Kg; i.p.) during the first week post-CCI. None of the doses of compound **23** caused significant pain diminution, whereas 50mg/kg was optimal for both EGCG and **30** to delay the latency of paw withdrawal. With 50 mg/Kg, we showed that EGCC prevented the thermal hyperalgesia from 7 to 21 dpi and compound **30** from 14 to 56 dpi. To evaluate the molecular mechanisms underpinning why EGCG and compound **30** differentially prevented the thermal hyperalgesia, we studied several biochemical parameters in the dorsal horn of the spinal cord at 14 and 56 dpi. We showed that the effect observed with EGCG and compound **30** was related to the inhibition of fatty acid synthase (FASN), a known target of these polyphenolic compounds. Additionally, we observed that EGCG and compound **30** reduced the expression of CCI-mediated inflammatory proteins and the nuclear localization of nuclear factor-kappa B at 14 dpi, but not at 56 dpi. We also strongly detected a decrease of synaptic plasma membrane levels of N-methyl-D-asparte receptor 2B in CCI-mice treated with compound **30** at 56 dpi. Altogether, compound **30** reduced the chronic thermal hyperalgesia induced by CCI better than the natural compound EGCG. Thus, our findings provide a rationale for the preclinical development of compound **30** as an agent to treat neuropathic pain.

## INTRODUCTION

Neuropathic pain is caused by injury or disease to the peripheral or central nervous system with no available effective treatment. After peripheral nerve injury, the characteristic features of neuropathic pain are spontaneous pain, hyperalgesia and allodynia [1]. These symptoms are caused by alteration of signaling pathways in neuronal populations located in the dorsal root ganglion, the spinal cord and cerebral areas [2]. Within the dorsal horn of the spinal cord, the activation of the second order sensory neurons contributed to the development and preservation of neuropathic pain [2], through the activation of several mechanisms, such as the production of inflammatory cytokines [3], the exacerbate activation of N-methyl-D-aspartate (NMDA) receptor [4] and the stimulation of transcriptional factors, such as the nuclear factor-kappa B (NF-κB) cascade [5]. Consequently, the development of new pharmacological agents with the capacity to interact with these spinal mechanisms could be a therapeutic solution for the neuropathic pain.

Epigallocatechin-3-gallate (EGCG), the main and most active catechin of green tea, is known to have therapeutic properties in many systems, included the nervous system. Some experimental works showed protective effects of EGCG against ischemia [6], neurodegenerative diseases [7, 8] and spinal cord injury [9–11]. Regarding neuropathic pain, few works have demonstrated an antinociceptive effect of EGCG. Recently, it has been reported in rats that intrathecal administration of EGCG attenuates mechanical allodynia and thermal hyperalgesia after chronic constriction nerve injury [12] and reduces mechanical allodynia after spinal nerve ligation [13]. These beneficial effects have been attributed to the antioxidant activity against nitric oxide [10, 13, 14] and reduction of pro-inflammatory cytokines expression [10, 12]. Other interesting targets have been described in non-neural cells, such as the enzyme fatty acid synthase (FASN). FASN catalyzes the synthesis of palmitate providing substrates to affect multiple cellular functions and it has been proposed as a therapeutic target in cancer [15]. Nevertheless, there are only few studies reporting the antinociceptive effect of EGCG at phases longer than two weeks after injury and its mechanism of action remains unclear. For instance, Renno *et al* [16] have recently shown that EGCG modulated chronic injured spinal cord in rats.

The therapeutic effect of EGCG has been well explored in cancer research showing some limitations for its *in vivo* effectiveness. EGCG exhibit a poor oral bioavailability, possibly due to the inability of EGCG to pass through the gut [17]. Furthermore, the EGCG is unstable because the hydroxyl groups could be modified [18] reducing its biological activity. To resolve these limitations, new polyphenolic compounds related to EGCG have been developed, showing higher therapeutic activity than EGCG [17, 19–21]. However, the effect of synthetic EGCG analogues has not been explored in nervous system-related disorders. Here, we compare the effects of EGCG and two polyphenolic derivatives related to EGCG (compounds **23** and **30**) in a mice model of neuropathic pain induced by chronic constriction injury of sciatic nerve. Moreover, we analyze in the dorsal horn of the spinal cord by which mechanism these polyphenolic compounds can exert their antinociceptive effect.

## MATERIAL AND METHODS

### Drugs and chemicals

The epigallocatechin-3-gallate (EGCG) was purchased from Sigma-Aldrich (St Louis, MI). The two synthetic EGCG derivatives: 1,5-Bis [(3,4,5-trihydroxybenzoyl) oxy] naphthalene (compound **23**) and 4,4′-Bis [(3,4,5-trihydroxybenzoyl) oxy]-1,1′-biphenyl (compound **30**) were synthesized as previously described [21]. The *Dc* protein assay kit was purchased from Bio-Rad Laboratories (Hercules, CA). The Total RNA isolation nucleo-spin RNA II kit was from Macherey-Nagel (Düren, Germany) and the StratraScript First Strand cDNA Synthesis System was purchased from Stratagene (Santa Clara, CA). The EC Western Blotting Detection Reagent was from Santa Cruz Biotechnology (Santa Cruz, CA) and the Hybond-C Extra Nitrocellulose membranes were purchased from Amersham (Little Chalfont, UK). All other chemicals were obtained from Sigma-Aldrich. Finally, the 6–0 poly-glycolic acid synthetic absorbable suture and 5–0 nylon suture were purchased from Suturas Aragó (Barcelona, Spain).

### Animals

Female Balb-c mice (21–22 g) were obtained from Charles River Laboratories (France). For the dose response study of EGCG and compounds **23** and **30**, 5 animals per group were used. To analyze the time-dependent effect of 50 mg/kg of EGCG and the compounds **23** and **30**, 15 animals per group were utilized. Another set of animals were used for the study at 14 days post-injury (dpi). In each case, 5 animals were utilized to study the fatty acid synthase activity, 5 animals for the PCR assays and 5 animals for the biochemical studies. At 14 and 56 dpi, mice were sacrificed with sodium pentobarbital (90 mg/kg; i.p.). Mice were housed with access to food and water *ad libitum* in a colony room kept at 19–22°C and 40–60% humidity, under a 12:12 hours light/dark cycle. All experimental procedures adhered to the recommendation of the European Union and the US Department of Health for the care and use of laboratory animals and were approved by the Ethics Committee of the Generalitat de Catalunya and Universitat de Barcelona (DARP6308).

### Chronic constriction injury

The chronic constriction injury (CCI) followed the procedure described elsewhere [22]. Animals were anesthetized with sodium pentobarbital (50 mg/kg; i.p.), an incision was made in the right thigh, and the sciatic nerve was exposed. Two loose ligatures with 1 mm apart were then made around the nerve using the 6–0 poly-glycolic acid synthetic absorbable sutures. The incision was closed using 5–0 interrupted nylon sutures. A control group was included with sham surgery where the right sciatic nerve was exposed but not further manipulated. The animals did not receive postoperative analgesics in order to preserve the pain associated with CCI and further to avoid undesirable interactions with EGCG or its derivatives.

### Pharmacological administration

For dose response studies, EGCG and the two synthetic EGCG derivatives compound **23** and compound **30** were administered by intra-peritoneal injection at 10, 30, 50 and 100 mg/Kg (n = 5 mice/compound/dose), 30 minutes after the CCI. To analyze the time-dependent effect EGCG and the two synthetic EGCG derivatives compound **23** and compound **30** were administered by intra-peritoneal injection at 50 mg/Kg (n = 15 mice/compound), 30 minutes after the CCI. Another set of animals were used to study the effect of 50 mg/kg of EGCG and the two synthetic EGCG derivatives compound **23** and compound **30** at 14 dpi (n = 15 mice/compound). In all cases, the administration was daily repeated during the first week. All compounds were dissolved in DMSO/saline solution (1:9; v/v), used as vehicle group.

### Thermal hyperalgesia

To measure changes in thermal sensation we used the plantar heat test (Ugo Basile, Italy) as previously described [23]. Briefly, mice were acclimated 5 days (15 min/day) prior to CCI. At 7, 14, 21, 28, 35, 42, 49 and 56 dpi, mice were enclosed in a clear Plexiglas box. After 15 min, during which the animals were provided with free exploration to habituate to the apparatus, infrared light beam was applied to the plantar surface of the forepaw, and paw withdrawal latency was recorded in seconds. The infrared stimulus application automatically shut off at 30 seconds to avoid tissue damage. Four trials were measured randomly for right forepaw with at least 2 minutes between each trial.

### Fatty acid synthase activity

The fatty acid synthase (FASN) activity was assayed in particle-free supernatants by recording spectrophotometrically at 37°C (Lambda Bio 20, Perkin Elmer Boston, MA, USA, EUA) and measuring the decrease of A340 nm due to oxidation of NADPH as previously described [24]. Briefly, mice (n = 5 per group) were sacrificed with sodium pentobarbital (90 mg/kg; i.p.) at 14 and 56 dpi. Spinal cord segments distal to T12 vertebra were quickly removed and snap frozen. The dorsal horn of the spinal cord tissue was homogenized in lysis buffer (250mM sucrose, 20 mM HEPES pH 7.6, 2 mM MgCl_2_, 1 mM DTT, 1 mM EDTA, 50 mM NaF, 2 mM phenylmethylsulfonyl fluoride (PMSF), 1 μg/μL aprotinin, 1 μg/μL leupeptin, 2 mM sodium orthovanadate). Homogenized samples were centrifuged at 3,500 x *g* for 10 minutes, and then supernatant was collected and centrifuged at 100,000 x *g* for 30 minutes to obtain supernatants particle-free. The protein concentrations were determined using the *Dc* protein assay kit. A 90 μg amount of protein was used for the reaction. A pre-incubation during 15 minutes at 37°C in 0.2 M of potassium phosphate buffer, pH = 7.0 was kept for temperature equilibration. Then, samples were added to the reaction mixture (200 mM potassium phosphate buffer, pH 7.0; 1 mM EDTA; 1 mM dithiothreitol, 30 μM Acetyl-CoA and 0.24 mM NADPH) and then were monitored at 340 nm for 3 minutes to measure background NADPH oxidation. After the addition of 50 μM of malonyl-CoA, the reaction was assayed for 10 minutes to determine FASN-dependent oxidation of NADPH. Rates were corrected with the background rate of NADPH oxidation.

### Quantitative (Q)-PCR assays

Total RNA was isolated from dorsal horn of spinal cord segments distal to T12 vertebra of all groups (n = 5 mice per group) using the Total RNA isolation nucleospin RNA II Kit. Purified RNA (600 ng) was reverse transcribed using the StrataScript First Strand cDNA Synthesis System. The cDNA synthesis was performed at 42°C for 60 minutes according to the manufacturer’s instructions. The cDNA was then analyzed by quantitative RT-PCR using the following TaqMan Gene Expression Assays (Applied Biosystems, Foster City, CA): 18S, HS99999901_s1; TNF-α, Mm00443258_m1; IL-1β, Mm00434228_m1; IL-6, Mm00446190_m1. The RT-PCR was performed as previously described [25]. Analysis and quantification was obtained with the comparative quantification analysis program of the Mx-Pro Q-PCR Analysis software version 3.0 (Strategene).

### Protein extraction and subcellular fractionation

Mice (n = 5 per group) were sacrificed with sodium pentobarbital (90 mg/kg; i.p.) at 14 and 56 dpi and spinal cord segments distal to T12 vertebra were quickly removed and frozen. For total protein extraction, the dorsal horn of the spinal cord was homogenized in modified RIPA buffer (50 mM Tris–HCl pH 7.5, 1% Triton X-100, 0.5% sodium deoxycholate, 0.2% SDS, 100 mM NaCl, 1 mM EDTA, 2 mM PMSF, 1 μg/μL aprotinin, 1 μg/μL leupeptin and 2 mM sodium orthovanadate).

Subcellular fractionation was obtained by differential centrifugation as described elsewhere [26]. Briefly, tissue was homogenized in lysis buffer (10 mM Tris-HCl pH 7.5, 1 mM EDTA, 1 mM Na_3_VO_4_, 0.25 M sucrose, 2 mM PMSF, 1 μg/μL aprotinin, 1 μg/μL leupeptin, 2 mM sodium orthovanadate). The homogenate was centrifuged at 2,000 × *g* for 10 min at 4°C to separate the nuclei fraction (P1). The P1 was resuspended in lysis buffer. The resulting supernatant was centrifuged at 10,000×*g* for 15 min at 4°C to obtain a crude membrane fraction (P2). The P2 was resuspended and incubated in the lysis buffer containing 0.5% Triton X-100 for 15 min, and then centrifuged at 25,000 × *g* for 20 min to obtain the synaptosomal membrane fraction (P3). Finally, the P3 was resuspended in lysis buffer and centrifuged on a discontinuous sucrose gradient (0.8 M, 1 M and 1.2 M) at 150,000×*g* for 2 h. Synaptic plasma membrane (SPM) was collected from the 1:1.2 M interface and resuspended in 50 mM HEPES, 2 mM EDTA, 1 mM PMSF, 10 μg/ml aprotinin, 1 μg/ml leupeptin and 2 mM sodium orthovanadate.

### Western blotting

Western blotting was performed as previously described [27]. The following primary antibodies were used: anti-FASN (1:1000, BD Bioscience, San Jose, CA); anti-TNF-α, anti-IL-1β, anti-IL-6 and anti-NFκB (all 1:500, Santa Cruz Biotechnology, Santa Cruz, CA); anti-phosphoNMDAR2B (Tyr1472) (1:2000, Affinity Bioreagents, Golden, CO) and anti-NMDAR2B (1:1000, Chemicon, Temecula, CA). Loading control was performed by reprobing the membranes with anti-actin (1:10,000; MP Biochemicals, Aurora, OH), anti-NeuN (1:1,000; Chemicon) or anti-N-cadherin (1:1000, BD Bioscience). Membranes were incubated with the corresponding horseradish peroxidase-conjugated antibody (1:2,000; Promega, Madison, WI). Immunoreactive bands were visualized using the Western Blotting Luminol Reagent and quantified by a computer-assisted densitometer (Gel-Pro Analyzer, version 4, Media Cybernetics).

### Statistical analysis

All data are expressed as mean ± SEM. All graphs were created with GraphPad Prism 4 Software Inc. version 4.02 (San Diego, CA). Different statistical analyses were performed as appropriate, and indicated in the figure legends. Values of *p*<0.05 were considered statistically significant.

## RESULTS

### Administration of EGCG and compound 30 differentially prevent the neuropathic pain induced by chronic constriction injury

In order to know whether EGCG and the two synthetic derivatives compounds **23** and **30** had the ability to prevent chronic neuropathic pain, we performed a dose-response study to test their effect on thermal sensation of forepaws in mice after chronic constriction injury (CCI). First, we daily intra-peritoneal (i.p.) administrated different doses of EGCG, compound **23** and **30** (10, 30, 50 and 100 mg/Kg) during the first week after CCI and we evaluated the latency to paw withdrawal to thermal sensation at 7 and 21 dpi. We observed that administration of 50 mg/Kg and 100 mg/kg of EGCG delayed the latency to paw withdrawal at 7 and 21 dpi, with best capacity at 50 mg/Kg (Table 1).

**Table 1:** Administration of EGCG and synthetic derivate compound 30 produce an increase in CCI-mediated thermal paw withdrawal latency in a dose- and time-dependent manner.

	10 mg/Kg	30 mg/Kg	50 mg/Kg	100 mg/Kg	
Vehicle	4.38±0.72				7 dpi
EGCG	3.93±0.33	5.01±0.35	13.01±1.02***	9.38±0.66***	
Compound **23**	4.07±0.31	4.57±0.29	4.38±0.3	4.41±0.27	
Compound **30**	3.71±0.39	3.93±0.37	4.31±0.3	4.79±0.44	
Vehicle	3.48±0.45				21 dpi
EGCG	3.83±0.23	4.28±0.45	8.58±0.9***	6.15±0.42***	
Compound **23**	3.89±0.43	3.77±0.59	3.93±0.24	3.71±0.29	
Compound **30**	4.11±0.51	4.33±0.57	7.61±0.67***	6.49±0.53***	

The latency to paw withdrawal to a thermal stimulus was recorded in CCI-mice treated with vehicle or different doses (10, 30, 50 and 100 mg/kg; i.p.) of EGCG, compound 23 and compound 30 at 7 and 21 days post injury (dpi). The results are expressed in seconds and data shown are the mean ± SEM (*n* = 5). Data were analyzed by two-way ANOVA with Bonferroni’s post-hoc test.

*** p<0.001 compared to vehicle-treated CCI-mice at any dpi.

**Fig 1:**
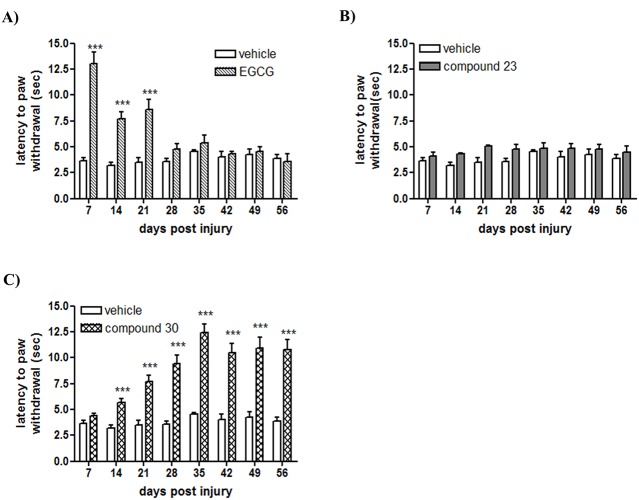
Administration of EGCG and compound 30 produce a time-dependent increase in CCI-mediated thermal paw withdrawal latency. The latency to paw withdrawal to a thermal stimulus was recorded in CCI-mice treated with vehicle and 50mg/Kg of EGCG (A), compound **23** (B) and compound **30** (C) from 7 to 56 days post injury. The results are expressed in seconds and data shown are the mean ± SEM (*n* = 15). Data were analyzed by two-way ANOVA with Bonferroni’s post-hoc test. *** p<0.001 compared to vehicle-treated CCI-mice.

In contrast, no statistically differences were observed at any dose between the group injected with vehicle and the groups injected with the compound **23** at 7 and 21 dpi (Table 1). Concerning the compound **30**, we detected a time-dependent effect at 50 and 100 mg/Kg. As we shown in Table 1, the administration of 50 mg/Kg and 100 mg/kg of compound **30** delayed the latency to paw withdrawal only at 21 dpi, with best effect at 50 mg/Kg. No statistically differences were observed at 10 and 30 mg/Kg (Table 1). As the best effect observed was 50 mg/Kg, we choose this concentration to evaluate whether the effect of polyphenolic compounds could be maintained at more chronic phases. Thus, with a new set of animals, we weekly assessed the thermal paw withdrawal latency in CCI-mice treated with 50 mg/Kg of EGCG, compound **23** and compound **30** from 7 to 56 dpi. Interestingly, we showed a time-dependent effect of EGCG and compound **30** in the prevention of neuropathic pain measured by thermal stimuli (Fig 1A and 1C). We observed an increase in paw withdrawal latency in CCI-mice treated with EGCG from 7 to 21 dpi, with a greater effect at 7 dpi (Fig 1A). In the other side, CCI-mice treated with the compound **30** resulted in an augmentation in the paw withdrawal latency from 14 to 56 dpi, with a highest effect from 35 to 56 dpi (Fig 1C). Concerning the compound **23**, we did not detect differences at any time point analyzed (Fig 1B). Stability studies of EGCG and compounds 23 and 30 showed that half-life of compound 23 in human and mouse serum is 1,4±0,3 and 0,3±0,1 hours, respectively and regarding compound 30, 1,9±0,2 and 0,4±0,3 hours in human and mouse serum (S1 Table). EGCG half-life in human and mouse serum is inferior to 0,1 hour. In serum, compound 23 and compound 30 are in equilibrium with their metabolites, which also displayed strong FASN activity inhibition (S1 Fig), in particular compound 30 (83% of FASN activity inhibition in SKBr3 human breast cancer cells). In addition, metabolites showed similar stability as their parental compounds 23 and 30 (data not shown).

### The prevention of CCI induced-neuropathic pain by EGCG and compound 30 is associated to a reduction of FASN activity in the dorsal horn of the spinal cord

**Fig 2:**
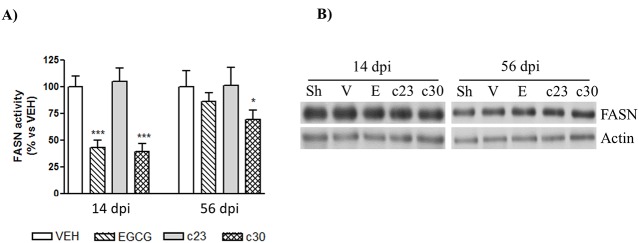
EGCG and compound 30 inhibit FASN activity in the spinal cord of CCI-mice. (A) The FASN activity was analyzed in the dorsal horn of the spinal cord of CCI-mice treated with vehicle, and 50 mg/Kg of EGCG, compound **23** (c23) and compound **30** (c30) as indicated in materials and methods at 14 and 56 days post injury (dpi). Data were expressed as a percentage with respect to vehicle-treated CCI-mice and represent the mean± SEM (*n* = 5). Data were analyzed by one-way ANOVA with Bonferroni’s post-hoc test. *p<0.05 and ***p<0.001 compared to vehicle-treated CCI-mice. (B) Protein extracts from the dorsal horn of the spinal cord of control mice (sham; Sh) and CCI-mice treated with vehicle (V), EGCG, compound **23** (c23) and compound **30** (c30) at 14 and 56 dpi were subjected to western blot to study the FASN protein levels. Representative immuno-blots showing no differences in FASN levels between all groups are presented. Actin levels were used as loading control.

It has been well reported the role of fatty acids in neuropathic pain after peripheral nerve injuries [28–30]. As it has been described in non-neural cells that EGCG inhibits the fatty acid synthase activity (FASN) [24], we studied the inhibition of FASN activity in the dorsal horn of the spinal cord of CCI-mice treated with EGCG and the two polyphenolic synthetic derivatives (compound **23** and compound **30**) at 14 (acute) and 56 (chronic) dpi. At 14 dpi, we observed that the administration of 50 mg/Kg of EGCG and the compound **30** reduced significantly the FASN activity to 57±8 and 61±8% of vehicle group (Fig 2A; n = 5). No differences were detected in CCI-mice administrated with the compound **23** (Fig 2A; n = 5). Importantly, the reduction of FASN activity at 56 dpi was only observed in CCI-mice treated with the compound **30** (Fig 2A; reduction of 32±9% of vehicle; n = 5). In order to know whether the decrease of FASN activity could be related to variations in FASN protein levels, we studied by western blot the protein levels of FASN in the dorsal horn of the spinal cord. EGCG, compound **23** and compound **30** had no effect on FASN protein levels in any group at any age (Fig 2B), indicating that the decrease of FASN activity was not caused by changes in FASN protein levels.

### The administration of EGCG and compound 30 produce an acute decrease of CCI-mediated inflammatory proteins in the dorsal horn of the spinal cord

**Fig 3:**
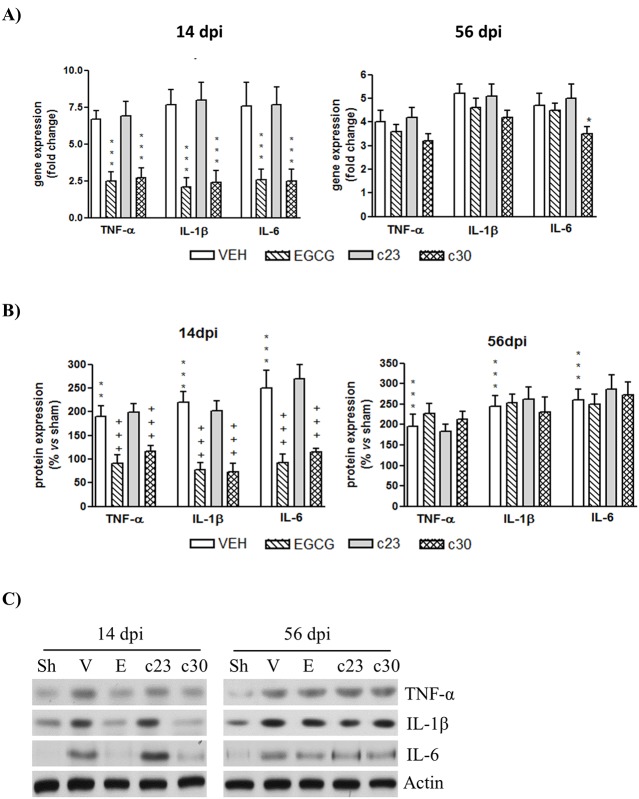
Spinal cord of CCI-mice treated with EGCG and compound 30 at 14 dpi showed decreased levels of inflammatory cytokines (A) Total RNA of the dorsal horn of spinal cord of control mice (sham; Sh) and CCI-mice treated with vehicle (VEH) and 50 mg/Kg of EGCG, compound **23** (c23) and compound **30** (c30) was isolated at 14 and 56 days post injury (dpi) as indicated in materials and methods. Quantitative RT-PCR analysis of TNF-α, IL-1β and IL-6 was performed. Data were expressed as fold change normalized to 18S and represent the mean± SEM (*n* = 5). * p<0.05 and *** p<0.001 compared to vehicle-treated CCI-mice using a one-way ANOVA with Bonferroni’s post-hoc test. (B) Protein extracts from the dorsal horn of the spinal cord of control mice (sham; Sh) and CCI-mice treated with vehicle (VEH) and 50 mg/Kg of EGCG, compound **23** (c23) and compound **30** (c30) at 14 and 56 days post injury (dpi) were subjected to western blot to study the TNF-α, IL-1β and IL-6 protein levels. Data were expressed as a percentage with respect to sham-mice. Results are the mean± SEM (*n* = 5) and represent the ratio between each protein and actin levels, obtained by densitometric analysis of western blot. Data were analyzed by one-way ANOVA with Bonferroni’s post-hoc test. *** p<0.001 compared to sham mice and ^+++^p<0.001 compared to vehicle-treated CCI-mice. (C) Representative immuno-blots are presented.

We next investigated whether the administration of polyphenolic compounds reverted the CCI mediated- expression of cytokines in the dorsal horn of the spinal cord. We explored by RT-PCR the mRNA expression of three cytokines related to neuropathic pain, tumor necrosis factor-α (TNF-α), interleukin-1 beta (IL-1β) and interleukin-6 (IL-6) at 14 and 56 dpi. As we show in Fig 3A, EGCG and compound **30** induced an important decrease in the mRNA levels of three cytokines at 14 dpi (approximately a 3-fold decrease compared to vehicle). In contrast no differences between vehicle and compound **23** were observed in any cytokine (Fig 3A). Surprisingly, this capacity of EGCG and compound **30** was not observed at 56 dpi (Fig 3A). Only a slight decrease in mRNA levels of IL-6 was detected in CCI-mice treated with compound **30** compared to CCI-mice with vehicle administration (Fig 3A). To know whether changes in mRNA levels implied variations in protein levels, we studied by Western blot the protein levels of TNF-α, IL-1β and IL-6. Accordingly to the mRNA levels, the protein levels of the three cytokines were reduced at 14 dpi in CCI-mice treated with EGCG and compound **30** compared to vehicle group (Fig 3B). At 56 dpi, no differences were detected between treated groups and vehicle group (Fig 3B).

**Fig 4:**
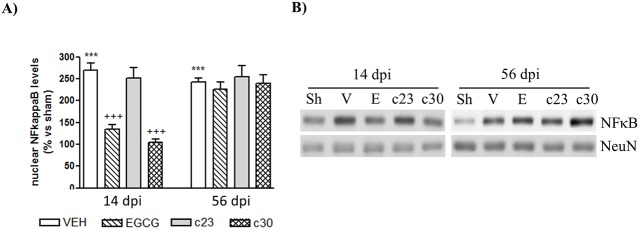
Reduced nuclear levels of NF-κB in the spinal cord CCI-mice administrated with EGCG and compound 30 at 14 dpi. (A) Nuclear protein extracts from the dorsal horn of the spinal cord of control mice (sham; Sh) and CCI-mice treated with vehicle (VEH) and 50 mg/Kg of EGCG, compound **23** (c23) and compound **30** (c30) at 14 and 56 days post injury (dpi) were subjected to western blot to study the NF-κB protein levels. Data were expressed as a percentage with respect sham-mice. Results are the mean± SEM (*n* = 5) and represent the ratio between each NF-κB and NeuN levels, obtained by densitometric analysis of western blot. Data were analyzed by one-way ANOVA with Bonferroni’s post-hoc test. ***p<0.001 compared to sham mice and ^+++^p<0.001 compared to and vehicle-treated CCI-mice. (B) Representative immuno-blots are presented.

The expression of cytokines in CCI-mediated neuropathic pain has been associated to activation of NF-κB [5]. So, we next studied the nuclear protein levels of NF-κB in the dorsal horn of spinal cord of CCI-mice treated with EGCG and the polyphenolic synthetic derivatives at 14 and 56 dpi. According to the cytokine levels observed in Fig 3, we showed that nuclear NF-κB levels are reduced only in CCI-mice treated with EGCG and compound **30** at 14 dpi (Fig 4A and 4B). There are no changes in CCI-mice treated with compound **23** at 14 dpi and in all treated groups at 56 dpi. All together our results suggest that the acute effect of EGCG and compound **30** against the neuropathic pain induced by CCI seems to be related to the reduction of inflammatory proteins in the dorsal horn of the spinal cord.

### The administration of compound 30 induce a decrease of synaptic plasma membrane levels of NMDAR2B in the dorsal horn of the spinal cord of CCI-mice

**Fig 5:**
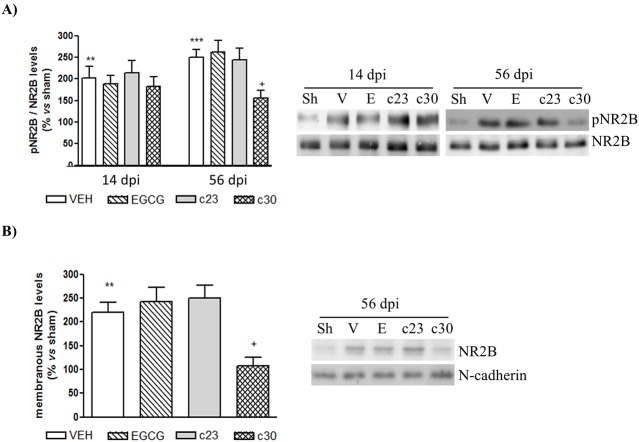
Decreased levels of NMDAR2B in the synaptic plasma membrane of spinal cord of CCI-mice treated with compound 30 at 56 dpi. (A) Protein extracts from the dorsal horn of the spinal cord of control mice (sham; sh) and CCI-mice treated with vehicle (VEH) and 50 mg/Kg of EGCG, compound **23** (c23) and compound **30** (c30) at 14 and 56 days post injury (dpi) were subjected to western blot to study the phospho-NMDAR2B and NMDAR2B protein levels. Data were expressed as a percentage with respect to sham-mice. Results are the mean± SEM (*n* = 5) and represent the ratio between phospho-NMDAR2B and NMDAR2B levels, obtained by densitometric analysis of western blot. Data were analyzed by one-way ANOVA with Bonferroni’s post-hoc test. **p<0.01 and ***p<0.001 compared to sham mice and ^**+**^p<0.05 compared to vehicle-treated CCI-mice. Representative immuno-blots are presented. (B) The synaptic plasma membrane of the dorsal horn of the spinal cord at 56 days post injury (dpi) of all groups was obtained as indicated in materials and methods and then subjected to Western blot to study the levels of NMDAR2B. Data were expressed as a percentage with respect to sham-mice. Results are the mean± SEM (*n* = 5) and represent the ratio between NMDAR2B and N-cadherin levels, obtained by densitometric analysis of western blot. Data were analyzed by one-way ANOVA with Bonferroni’s post-hoc test. **p<0.01 compared to sham mice and ^+^p<0.05 compared to vehicle-treated CCI-mice. Representative immuno-blots are presented.

It has been involved an enhancement of N-methyl-D-aspartate receptor (NMDAR), through the expression of NMDAR2B subunit, in the dorsal horn of spinal cord with the induction of neuropathic pain [4]. To know whether the administration of EGCG or the two polyphenolic synthetic derivatives had the capacity to prevent the activation of NMDAR, we analyzed the phosphorylated levels of NMDAR2B subunit in the dorsal horn of spinal cord at 14 and 56 dpi. At 14 dpi, there were no changes in the phospho-levels of NMDAR2B between vehicle group and treated groups (Fig 5A). Interestingly, although the treatment with EGCG and compound **23** did not induce variations in the phospho-levels of NMDAR2B at 56 dpi, we detected a specific decrease of the phosphorylation of NMDAR2B in CCI-mice treated with compound **30** compared to vehicle group (Fig 5A). No significant changes were observed in the total levels of NMDAR2B in any group (Fig 5A). Variations in the levels of tyrosine phosphorylation of NMDAR2B could be related to a stable surface expression through palmitoylation [31]. To know whether the decrease of phospho-NMDAR2B observed in CCI-mice treated with compound **30** was associated with a reduction in the surface membrane expression, we analyzed by Western blot the levels of NMDAR2B in a synaptic plasma membrane (SPM) fraction of the dorsal horn of the spinal cord at 56 dpi. We strongly detected a specific decrease in the protein levels of NMDAR2B in the SPM of CCI-mice treated with compound **30** (Fig 5B). The administration of EGCG and compound **23** did not result in changes in NMDAR2B levels in the SPM fractions (Fig 5B). These results suggest that chronic prevention of CCI-induced neuropathic pain by compound **30** is associated to a reduction of NMDAR2B levels in the synaptic fractions of the dorsal horn of the spinal cord.

## DISCUSSION

The clinical management to treat neuropathic pain is pharmacological therapy, however nowadays there is no effective treatment [32, 33]. Here, we showed that the synthetic polyphenol related to EGCG, compound **30**, was significantly more effective than EGCG in reducing thermal hyperalgesia in a chronic CCI-mice model. While EGCG reduced the thermal hyperalgesia up to 21 dpi, compound **30** was able to decrease the nociceptive stimulus to 56 dpi. The antinociceptive effect of EGCG in neuropathic pain models has been previously described in acute [12, 13] and chronic [16] phases. Here we showed that analgesic capacity of EGCG failed to be effective at chronic stages. In contrast, we provide data showing that compound **30** had the ability to extend the antinociceptive effect until chronic phases. EGCG exhibits poor oral bioavailability due to poor absorption and biotransformation reactions. Numerous alterations to EGCG molecule have been described either to improve the integrity of the native compound or to generate more stable yet similarly efficacious molecules [16, 17]. Our group and others have produced the synthesis of new polyphenolic derivatives (compound **23** and compound **30**, among others) related to EGCG as anti-cancer agents, improving the *in vivo* bioavailability and efficacy of EGCG [17, 19, 20, 21]. Stability studies in human and mouse serum showed that compound **23** and **30** improved half-life and displayed strongest FASN activity inhibition respect their parental compound, EGCG. In addition, as we previously published, toxicity studies of compound **23** and compound **30** showed that neither **23** nor **30** caused changes on body weight *versus* the control group [19, 21, 24]. Food and fluid intake were similar to controls, and altered behavior or signs of suffering or distress were not observed in mice treated with **23** and **30** (data not shown). Hepatic and renal function serum markers (aspartate transaminase, alanine transaminase, alkaline phosphatase, creatinin and urea) and hematological function serum markers (% neutrophils, % lymphocytes, % monocytes, % platelet cells % hematocrit and hemoglobin) showed no significant alteration between control and experimental animals treated with compound **23** at daily doses of 5, 25 or 40 mg/Kg, as we previously reported [21]. Regarding compound **30**, no significant alteration in hepatic, renal and hematological function serum markers between control and experimental animals treated at daily doses of 50 and 75 mg/Kg were detected (see S2 Table). Furthermore, histological examination of liver, heart, kidney lung and brain showed no microscopic evidence of drug induces toxicity in compound **23**- and **30**- animals when compared with control animals (data not shown). In present study found that body weight loss was less than 1% in animals treated with polyphenols. This pattern also was observed in control mice (data not shown). Here, we show for the first time the beneficial effect of an EGCG derivative in neuropathic pain. Different clinical trials in neurodegenerative disorders has been developed using EGCG thus, we thought that our findings provide a rationale for the preclinical development of compound **30** as an agent to treat neuropathic pain.

We observed that the effect of EGCG and compound **30** on thermal hyperalgesia is strongly correlated with the inhibition of FASN activity in the dorsal horn of the spinal cord. These findings suggest that in the nervous system, the therapeutic effect of EGCG and compound **30** is mediated by the reduction of FASN activity, as we and others previously reported in cancer models [15, 24]. In addition, the polyphenolic derivative with no action on nociceptive stimulus, compound **23**, was not a potent FASN activity inhibitor. Accordingly, we have previously reported the different % of FASN inhibition of compound **23** and **30** (30% *versus* 90% respectively) [21]. FASN mainly synthesize the fatty acid palmitate [34]. Palmitate is implicated in the S-palmitoylation of several proteins altering their activity [35, 36]. Some of them are directly related to the generation of neuropathic pain in the dorsal horn of the spinal cord: (i) the activity and expression of pro-inflammatory enzymes is related to palmitoylation [37–40]; (ii) palmitate also activates the NF-κB transcription factor and induces the expression of pro-inflammatory cytokines and chemokines in several cells such as adipocytes [41], and muscle cells [42, 43]; and (iii) palmitoylation regulates the expression and clustering of NR2A and NR2B subunits of NMDA receptors [44, 45]. Altogether, FASN seems to be an enzyme with capacity to regulate several spinal mechanisms related to neuropathic pain and we highlighted FASN as an attractive therapeutic target to reduce the neuropathic pain.

Our study revealed that the acute analgesic effect (14 dpi) of EGCG and compound **30** is associated with the reduction of the expression of pro-inflammatory cytokines. However, in chronic phases (56 dpi) another molecular mechanism is involved in this setting. Accordingly, we observed the same pattern with the nuclear levels of NF-κB. The synthesis of the pro-inflammatory cytokines TNF-α, IL-1β and IL-6 is mediated by the nuclear activation of NF-κB, which may play a pivotal role in neuroinflammation [46]. It has been reported that intrathecal injection of antisense oligonucleotides directed to p65 subunit of NF-κB alleviates neuropathic pain after chronic constriction injury of the sciatic nerve [47]. Moreover, the increased expression of pro-inflammatory cytokines in neuropathic pain is considered the main cause of hyperalgesia [48]. Our results are in accordance with Kuang *et al* [12] showing that EGCG reduced thermal hyperalgesia and mechanical allodynia through the decreasing of the activity of NF-κB and the reduction of the synthesis of pro-inflammatory cytokines such as TNF-α and IL-1β. Here, we prove that the capacity of EGCG and compound **30** reduced the neuroinflammation until at least 14 dpi and then disappeared at chronic stages. Accordingly there is previous report showing that neuropathic pain was associated with the synthesis of pro-inflammatory cytokines at early stages but not at later stages [48] suggesting the involvement of other mechanisms into developing neuropathic pain at later stages.

Importantly, we associated the antinociceptive effect of compound **30** at chronic phases with a reduction of NMDA receptor activity. We detected a specific decrease in the phosphorylation of NR2B protein levels in CCI-mice treated with compound **30** at 56 dpi. We showed that the reduction of phospho-levels of NR2B was associated with a decrease in the protein levels in the synaptic plasma membrane. It is well known the involvement of the NMDA receptor activation in the dorsal horn of the spinal cord for the preservation of neuropathic pain [4]. Interestingly, it has been described that the expression of NR2B is restricted to the dorsal horn of spinal cord [49], and notably in the lamina II [50]. The increase of this subunit is involved in long-term plastic changes that occur in spinal cord [50] that favors the sensitization of nociceptive neurons located in the dorsal horn of the spinal cord [51]. The potentiation of this sensory response enhances the chronic hyperalgesia [2, 52]. The posttranslational palmitoylation of NR2B subunit regulates localization and activity of NMDA receptor [51]. So, it is tempting to speculate that prolonged inhibition of FASN by compound **30** diminishes the expression of NR2B in the synaptic plasma membrane, reducing the chronic sensory response that induce thermal hyperalgesia.

Taken together, our results showed that compound **30**, but not EGCG and compound **23**, prolonged the analgesic effect of EGCG in chronic phases. We associated this effect to the inhibition of the enzyme fatty acid synthase (FASN) by compound **30**. Moreover, we showed evidences that the antinociceptive effect of compound **30** was mediated by different molecular mechanisms in the dorsal horn of the spinal cord. In acute phases, compound **30** displayed their effect reducing neuroinflammation and in chronic stages the effect was through regulating NR2B expression in the synaptic plasma membrane. In summary, our findings provide a rationale for further pre-clinical investigation on the therapeutic regimen based on FASN inhibitors in neuropathic pain refractory to current therapies.

## Supporting Information

S1 Fig Pharmacological properties of compound 23 and compound 30 metabolism. (TIF)

S1 File Serum Stability Study. (DOCX)

S1 Table Half-life of EGCG and compounds 23 and 30 in human and mouse. (DOCX)

S2 Table Hepatic, renal and hematological function serum markers of compound 30-treated Balb/c. (DOCX)
