# Supplementary figures and images for: Novel Epigallocatechin-3-Gallate (EGCG) Derivative as a New Therapeutic Strategy for Reducing Neuropathic Pain after Chronic Constriction Nerve Injury in Mice
Source: PLoS One. 2015 Apr 9;10(4):e0123122. doi: 10.1371/journal.pone.0123122 (PMC4391943; doi:10.1371/journal.pone.0123122)

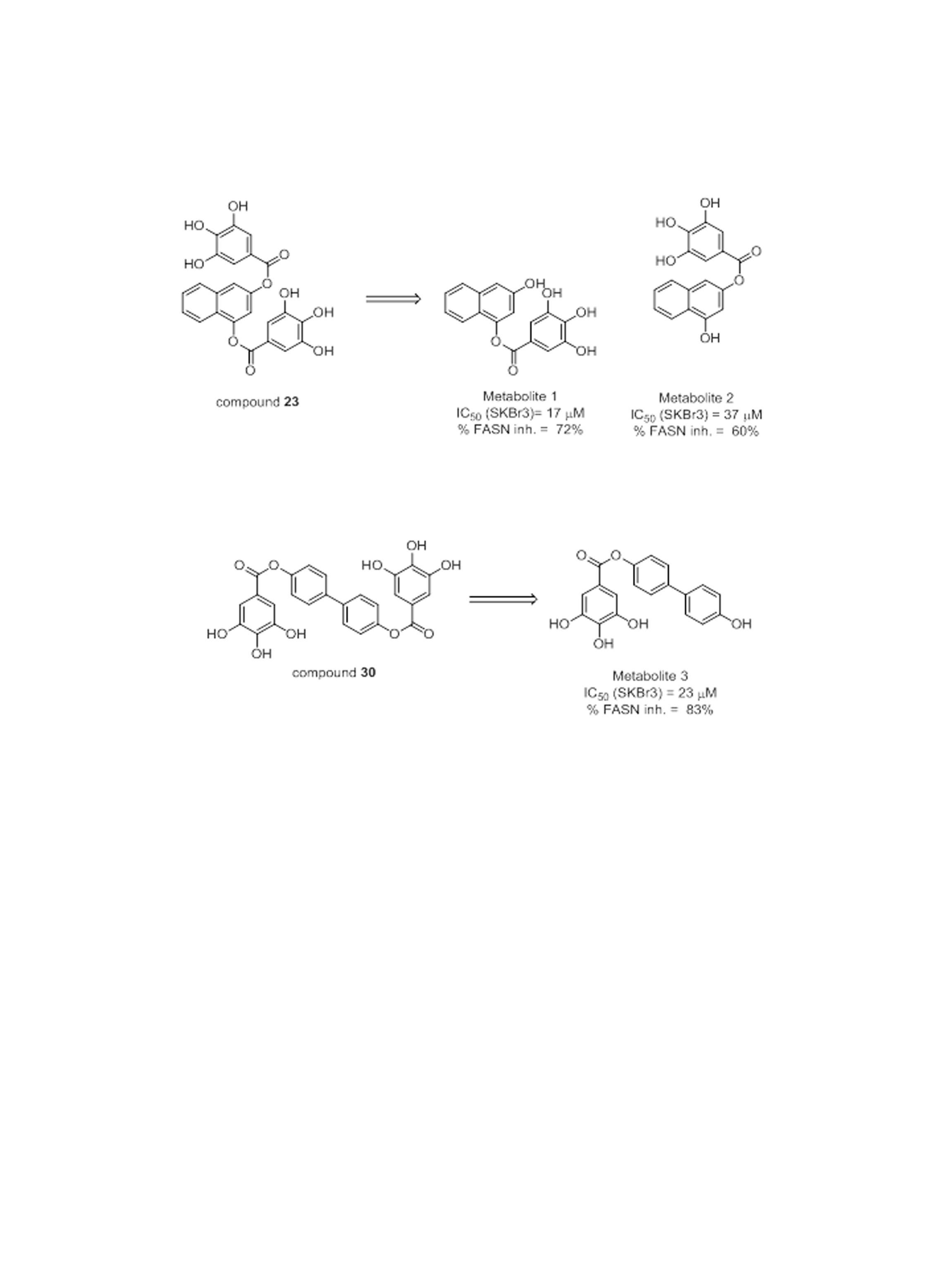

Supplement: S1 Fig — (TIF) [file pone.0123122.s001.tif]
