# Supplementary material for: Novel Epigallocatechin-3-Gallate (EGCG) Derivative as a New Therapeutic Strategy for Reducing Neuropathic Pain after Chronic Constriction Nerve Injury in Mice
Source: PLoS One. 2015 Apr 9;10(4):e0123122. doi: 10.1371/journal.pone.0123122 (PMC4391943; doi:10.1371/journal.pone.0123122)
Supplement: S1 File — (DOCX) [file pone.0123122.s002.docx]

Peptide stabilities were assayed in diluted serum as previously described. Human serum (Sigma, S7023) or mouse serum (Europa Bioproducts, EQSM-0100) were incubated at 37°C for 10 min. The assay was initiated upon the addition of the test compound solved in PBS to the serum (75% final serum content) for a final compound concentration of 0.5 mM. Then, 500 µL aliquots of the incubations were taken for the following time points: 0, 15, 30, 60, 120 and 240 min. The aliquots were mixed with 750 µL of cold acetonitrile (ACN) and incubated at 4°C for 10 min to precipitate serum proteins. The supernatant was collected for each sample after centrifugation at 39,000 *g* for 10 min and were immediately analyzed by high performance liquid chromatography-mass spectrometry (HPLC-MS).

High pressure liquid chromatography−mass spectrometry (HPLC-MS) analysis was performed using an Agilent 1200LC-MSD VL. LC separation was achieved with an Eclipse XDB-C18 column (5 μm, 4.6 mm × 150 mm) together with a guard column (5 μm, 4.6 mm × 12.5 mm). The gradient mobile phases consisted of A (95:5 water/MeOH) and B (5:95 water/MeOH) with 0.1% ammonium hydroxide and 0.1% formic acid as the solvent modifiers. The used gradient was: 2 min of isocratic 100% phase A, linear gradient to 60% of phase B for 6 min, then a second linear gradient to 100% of phase B for 12 min. An isocratic 100% phase B for 5 min and, finally the column was equilibrated using 100% phase A for 5 min. A flowrate of 0.5 mL/min was used for the entire gradient. MS analysis was performed with an ESI source set with the selected ion monitoring (SIM) mode. The capillary voltage was set to 3.0 kV, and the fragmentor voltage was set at 72 eV. The drying gas temperature was 350 °C, the drying gas flow was 10 L/min, and the nebulizer pressure was 20 psi.
