# Supplementary material for: Novel Epigallocatechin-3-Gallate (EGCG) Derivative as a New Therapeutic Strategy for Reducing Neuropathic Pain after Chronic Constriction Nerve Injury in Mice
Source: PLoS One. 2015 Apr 9;10(4):e0123122. doi: 10.1371/journal.pone.0123122 (PMC4391943; doi:10.1371/journal.pone.0123122)
Supplement: S1 Table — (DOCX) [file pone.0123122.s003.docx]

| **Compound** | **Serum** | **t_1/2_ (h)^a^** |
| --- | --- | --- |
| Compound **23** | Human | 1.4 ± 0.3 |
|  | Balb/c | 0.3 ± 0.1 |
| Compound **30** | Human | 1.9 ± 0.2 |
|  | Balb/c | 0.4 ± 0.2 |
| EGCG | Human | < 0.10 |
|  | Balb/c | < 0.10 |

Half-life (t_1/2_) mean ± E.E. Values were obtained from two independent experiments.
