# Supplementary material for: Novel Epigallocatechin-3-Gallate (EGCG) Derivative as a New Therapeutic Strategy for Reducing Neuropathic Pain after Chronic Constriction Nerve Injury in Mice
Source: PLoS One. 2015 Apr 9;10(4):e0123122. doi: 10.1371/journal.pone.0123122 (PMC4391943; doi:10.1371/journal.pone.0123122)
Supplement: S2 Table — (DOCX) [file pone.0123122.s004.docx]

|  | **Renal Function**  **(mg/dl)** | | **Hepatic Function**  **(U/L)** | | | **Haematological Parameters (%)** | | | | | |
| --- | --- | --- | --- | --- | --- | --- | --- | --- | --- | --- | --- |
|  | **BUN^*^** | **Creatinin** | **ALT^†^** | **ALP^‡^** | **AST^§^** | **Neutrophils** | **Lymphocytes** | **Monocytes** | **Platelet cells** | **Hematocrit** | **Hemoglobin (g/dl)** |
| **Vehicle**  **Control** | 29.1 + 5.4 | 0.32 + 0.1 | 198.7 + 22.5 | 92.5  + 25.8 | 990.3 + 40.6 | 28.9 + 7.7 | 62.6 + 5.7 | 2.1 + 0.6 | 854.7 + 206.1 | 38.7 + 4.6 | 13.3 + 1.0 |
| **Compound 30**  **50 mg/Kg** | 21.9 + 7.5 | 0.30+ 0.1 | 210.3 + 28.2 | 97.2 + 20.3 | 266.5 + 80.3 | 32.0 + 22.9 | 51.2 + 13.9 | 1.4 + 0.1 | 826.5 + 67.2 | 42.4 + 2.2 | 14.2 + 0.8 |
| **Compound 30**  **75 mg/Kg** | 21.1 + 6.8 | 0.31 + 0.1 | 290.2 + 31 | 86.5 + 30.3 | 320.3 + 45.5 | 30.7 + 8.1 | 50.2 + 7.5 | 1.9 + 1.4 | 849.0 + 65.0 | 38.3 + 2.8 | 13.6 + 0.3 |

Hepatic, renal and hematological function serum markers of compound **30**-treated Balb/c***** ^*^BUN: urea; ^†^ALT: Alanine transaminase; ^‡^ALP: Alkaline phospatase; ^§^AST: Aspartate transaminase; ^**^ (p<0.05) indicate the level of statistical significance compared to vehicle control.
